# Predictive role of a combined model for futile recanalization in acute ischemic stroke: a retrospective cohort study

**DOI:** 10.3389/fneur.2025.1566842

**Published:** 2025-05-15

**Authors:** Yangbin Zhou, Yitao Zhou, Huijie Yang, Xiaoyan Wang, Xiping Zhang, Ganying Huang

**Affiliations:** ^1^School of Nursing, Zhejiang Chinese Medical University, Hangzhou, China; ^2^Department of Emergency, Afliated Hangzhou First People’s Hospital, School of Medicine, Westlake University, Hangzhou, Zhejiang, China

**Keywords:** futile recanalization, acute ischemic stroke, ROC curve, mechanical thrombectomy, AIS-LVO

## Abstract

**Objective:**

There is a lack of data regarding patients with acute ischemic stroke caused by large vessel occlusions (LVOs) undergoing mechanical thrombectomy (MT) and their predictors of futile recanalization (FR). We sought to investigate the predictors of FR in patients with AIS-LVO undergoing mechanical thrombectomy.

**Method:**

A retrospective analysis was conducted on 229 acute AIS patients who received MT, after eliminating the 31 patients not meet the requirements. The patients were categorized into the FR group and the useful recanalization (UR) group. Multivariate logistic regression analysis was used to explore the factors that influence FR after mechanical thrombectomy. ROC curve was used to plot the ability to predict FR after MT, and then the combined model was constructed and evaluate the predictive ability of this model to FR.

**Results:**

198 patients who achieved successful recanalization were included in the analysis, of whom 124 experienced UR and 74 experienced FR. Patients with FR had higher Baseline NIHSS; they were more frequently on hypertension history and had longer door-to-puncture time (DPT) and door-to-recanalization time (DRT). Multivariable regression analysis showed that the hypertension history, Admission NIHSS, Admission DBP, Admission blood glucose, ischemic core, and DPT were associated with an increased probability of FR. The combined model was better than the models alone in predicting the risk of FR.

**Conclusion:**

Admission blood pressure, admission NIHSS scores, admission DBP, ischemic core and DPT are independent risk factors for FR after MT in patients with AIS, and the combined model established by them has high predictive efficacy for FR risk after MT.

## Introduction

As the second-leading cause of death disease, Stroke is a widespread neurological condition and the primary cause of disability worldwide (1). Also, it resulted in approximately 6 million annual fatalities. Ischemic stroke accounts for 71% of all strokes worldwide and 81.9% in China. The proportion of acute ischemic stroke (AIS) caused by large vessel occlusions (LVOs) in Chia was 20% (2). Acute ischemic stroke (AIS) is a sudden neurologic dysfunction caused by focal brain ischemia which is accompanied by imaging evidence of acute infarction (3). AIS occur caused by focal cerebral hypoperfusion, particularly from embolism and atherosclerotic disease. At present, the main effective treatment method for early reperfusion in acute ischemic stroke is intravenous rt-PA thrombolysis (4–6). For AIS-LVOs, the vascular revascularization rate of intravenous thrombolysis is low (13% ~ 18%) and the therapeutic effect is not good (7). The successful recanalization rate of MT has achieved 41–88%, which was much higher than that yielded by traditional therapies, including intravenous thrombolysis (8). Partial randomized clinical trials (RCTs) (9–14) have proven benefits on functional outcomes of endovascular thrombectomy (EVT) compared with intravenous thrombolysis. The functional outcomes of AIS patients with proximal anterior circulation LVO were improved by MT, particularly in those with good collateral circulation (6, 15). Preceding randomized controlled trials (10–12, 14, 16, 17) have consistently demonstrated that, among patients receiving standard care, MT markedly enhances successful reperfusion.

The modified Thrombolysis in Cerebral Infarction (mTICI) score can evaluate the degree of recanalization, which is considered a powerful predictor of good functional prognosis (18, 19). However, FR are not always associated with successful or complete reperfusion. Previous studies have revealed that more than 50% of patients suffer from futile recanalization (FR), which is defined as an adverse functional outcome at 90 days despite successful recanalization (mTICI = 2b-3) (18, 20). FR was linked to age, admission NIHSS, comorbidities, Alberta Stroke Program Early CT Score (ASPECTS), as well as time from symptom onset to recanalization (21, 22). Furthermore, studies have demonstrated that a high mRS score prior to stroke onset, coexisting dyslipidemia, and atrial fibrillation were identified as predictors of FR (23).

Therefore, it is of paramount importance to better understand the therapeutic effect of patients after MT and determine the factors that may help predict the occurrence of FR in patients. Predictive models for the occurrence of FR following MT surgery in patients are relatively scarce. Such models are necessary to accurately convey potential risks and benefits to the patients themselves or the proxies, and facilitate patient-oriented informed decision-making. The advent of reliable prediction models is capable of adapting to the continuously escalating healthcare demands and costs in China.

We conducted an observational retrospective study aiming to explore the predictors of futile recanalization in patients with LVO undergoing MT. Therefore, this study aims to utilize the National Stroke Center Construction Management Information System (NSCCMI) registry to clarify the predictive ability of admission blood pressure, baseline NIHSS scores, admission DBP, ischemic core and DPT for the risk of FR after MT in patients with AIS.

## Methods

### Study design and participants

The cohort was comprised of patients enrolled in the NSCCMI registry (National Stroke Center Construction Management Information System), a cohort study registering AIS patients in China which includes a hospital-based follow-up study. We enrolled 229 AIS-LVO patients from Hangzhou First People’s Hospital who were treated with mechanical thrombectomy between March 2022 and February 2024. The sample size met the principle of 10 Events Per Variable (EPV) (24). Inclusion criteria were 198 patients who achieved successful recanalization were included in the analysis, of whom 124 (62.63%) experienced UR and 74 (37.37%) experienced FR. The sample size met the principle of 10 Events Per Variable (EPV). All participating subcenters were obligated to recruit consecutive patients, and all patients or their legal representatives supplied informed consent. All patients used computed tomography and/or magnetic resonance imaging to diagnose AIS. According to TOAST criteria (25), AIS can be divided into four subtypes: (1) large-artery atherosclerosis (LAA), (2) cardioembolism (CE), (3) small-artery occlusion, (SAO), (4) stroke of other determined etiology, and (5) stroke of undetermined etiology (25). Categories 4 to 5 were defined as “other causes” in this study. This study included patients with subtypes according to TOAST criteria. All patients were followed for 3 months after AIS onset.

The present study enrolled patients with AIS-LVO undergoing MT between March 2022 and February 2024. Patients met the following inclusion criteria: (1) Age 18–90 years; (2) meet the diagnostic criteria for AIS (26); (3) patients treated with MT; (4) mTICI of 2b-3 after MT (27); (5) without rheumatoid immune disorders, severe hepatic or renal disorders, hematological disorders, or malignant tumors; (6) without any systemic infections that occurred at the time of specimen collection or 2 weeks before stroke onset; (7) finish 90-day follow-up.

MT was selected for patients meeting the following criteria: (1) confirmed AIS, and bleeding or other pathological brain diseases ruled out by CT; (2) LVO confirmed by CTA or digital subtraction angiography; (3) MT treatment can be initiated between 6 and 16 h of stroke onset (28); (4) obtaining informed consent from family members. Exclusion criteria: (1) confirmed intracranial hemorrhage or intracranial tumor on admission; (2) inability to take care of oneself; (3) previous psychiatric disorders that would interfere with neurologic evaluation; (4) Other serious, advanced, or terminal illness (investigator judgment) or life expectancy is less than 6 months; (5) Any other condition that, in the investigator’s judgment, precludes an endovascular procedure or poses a considerable risk to the subject in the event that an endovascular procedure is performed; (6) incomplete baseline data.

### Data collection

**Figure 1:**
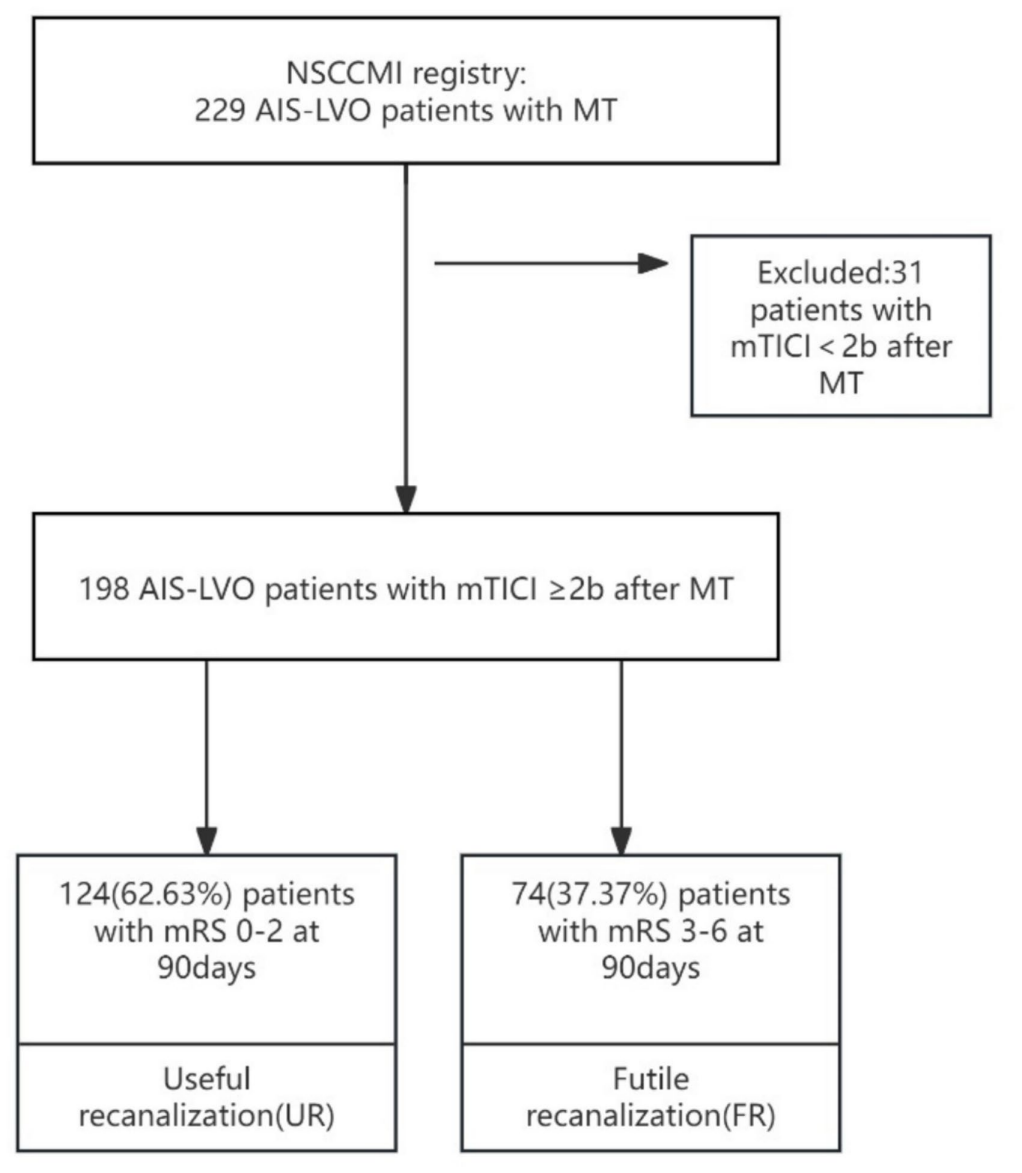
Study flow chart.

**Figure 2:**
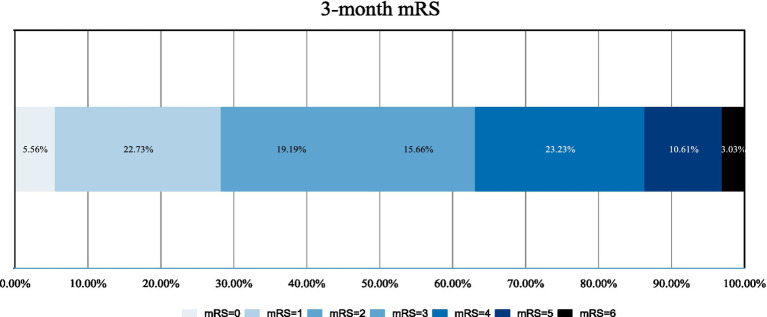
Distribution of mRS at 90 days among included patients. mRS modified Rankin Score.

Baseline assessment, including demographic features, stroke risk factors, admission NIHSS, admission blood glucose, admission blood pressure (SBP and DBP), DPT (door-to-puncture time), OPT (stroke onset-to-puncture time), DRT (door-to-recanalization time), ischemic penumbra, ischemic core, and other laboratory data were recorded. The modified Rankin Scale (mRS) was utilized to evaluate the patient’s stroke status after 90 days. Two independent and trained raters for each center, who were not involved in the endovascular stroke treatment of the included patients and were oblivious to any clinical and treatment information, assessed the modified Rankin Scale (mRS) of the patients at 90 days centrally either through a telemedicine consultation or in-person consultation. Any discordance was resolved with the participation of a senior stroke neurologist for each center as a third party not involved in the care of the patients. The investigators received training and obtained qualification certificates for recording the National Institutes of Health Stroke Scale (NIHSS) and mRS. NIHSS was carried out in all patients on admission (Figures 1, 2).

### Definition of futile recanalization

We defined FR as AIS patients experiencing a 90-day poor outcome (mRS 3–6) despite successful recanalization (mTICI ≥ 2b) after MT, and useful recanalization (UR) defined as AIS patients achieving a 90-day good outcome (mRS ≤ 2) with successful recanalization after MT.

### Statistical analysis

Statistical analyses were performed using IBM SPSS 22.0 and R. We used descriptive statistics to summarize the variables collected.

Statistical analysis was performed using SPSS 27.0 software. The Shapiro–Wilk test was used to assess the normality of quantitative data. Normally distributed continuous variables were presented as mean ± standard deviation (x ± s), and the independent samples t-test was used to compare the two groups. Non-normally distributed continuous variables were presented as median (Q1, Q3), and the Mann–Whitney U test was used to compare the two groups. Categorical variables were presented as frequency (%), and the *χ*^2^ test or Fisher’s exact test was used to compare the two groups. Multivariate logistic regression analysis was then conducted to identify independent risk factors for FR in AIS from large-vessel occlusion after MT. Multivariate models included Hypertension, Admission NIHSS, Admission DBP, Admission blood glucose and ischemic core.

**Figure 3:**
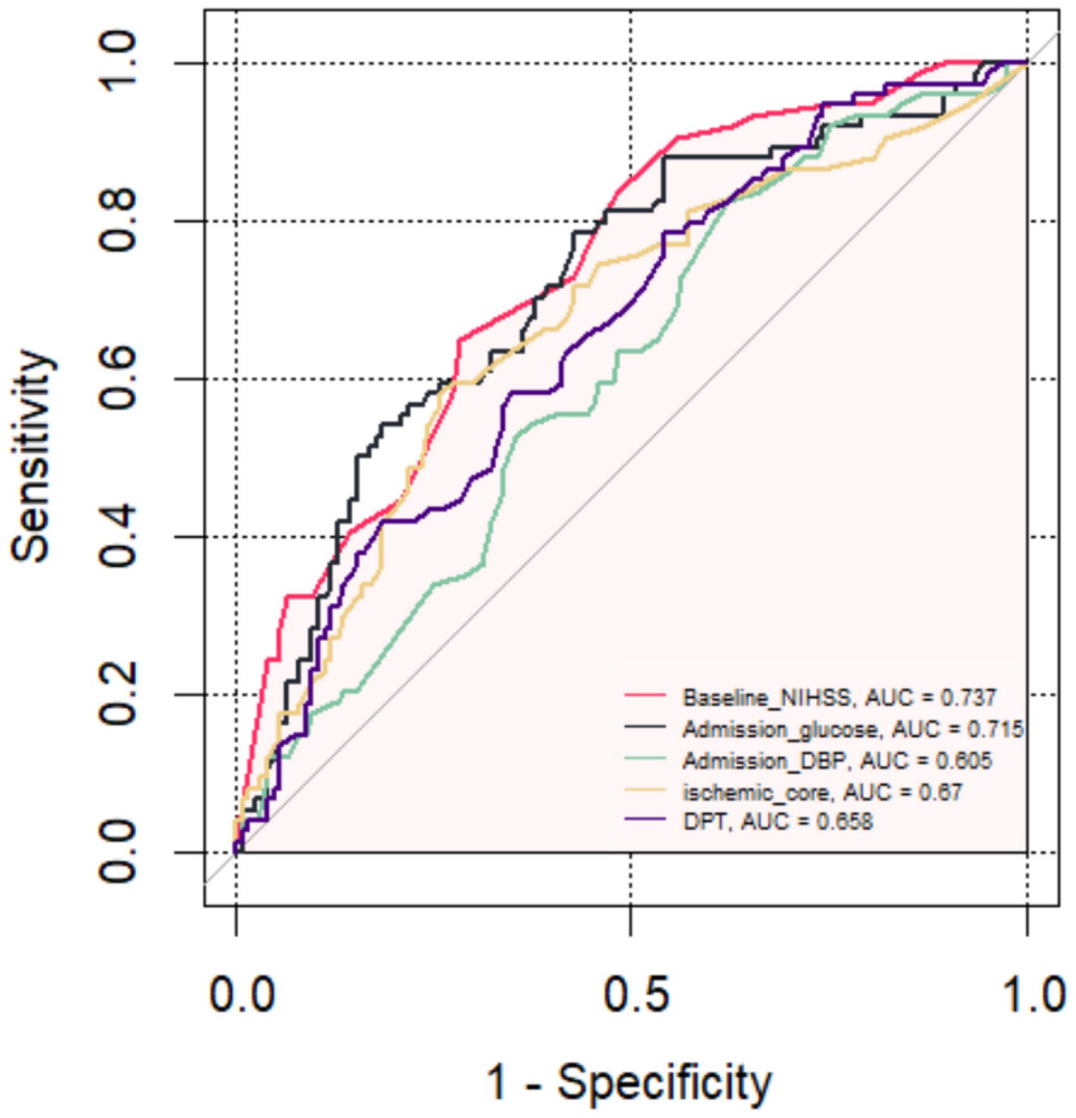
The receiver operating characteristic curve of the five main predictors for Futile Recanalization.

**Figure 4:**
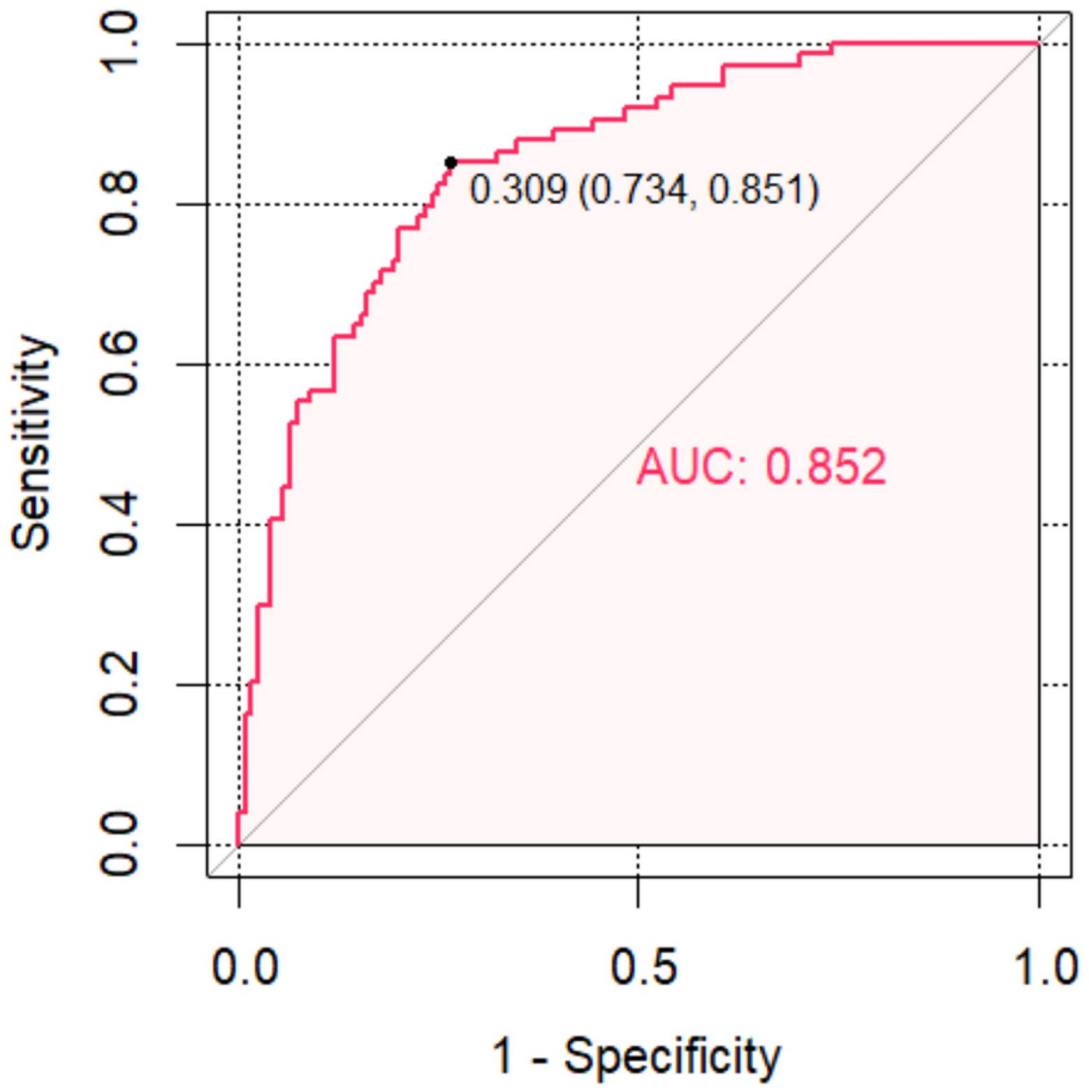
The receiver operating characteristic curve of the Combined model for Futile Recanalization.

The relationship between the risk factors and the probability of poor outcome, as well as the odds ratio (OR), was assessed. Finally, receiver operating characteristic (ROC) curves were constructed to evaluate the predictive ability of the risk factors and other indicators for FR by comparing the area under the curve (AUC). A significance level of *p* < 0.05 was considered statistically significant (Figures 3, 4).

## Results

### Comparison of clinical data

A total of 198 patients were included in the study, with 124 cases in the UR group (76 males and 48 females) and 74 cases in the FR group (44 males and 30 females). The mean (SD) age was 70.54 ± 12.12 years, and 60.6% of the participants were males. There were no significant differences between the two groups in terms of gender, age, drinking history, smoking history, or other baseline characteristics (all *p* > 0.05).

However, the proportion of patients has a hypertension history in the FR group was significantly higher than in the UR group (*p* < 0.05) (Table 1). The FR group had a significantly longer with DPT [97 (79.75, 125.25) vs. 83 (51.5, 102.75), *p* = 0.001], DRT [137 (105.75, 178.5) vs. 116 (83.25, 144) *p* = 0.003], higher baseline NIHSS scores [20 [17.0–29.0] vs. 15.0 [11.0–20.0] *p* < 0.001], admission DBP [93.64 ± 17.18 vs. 87.10 ± 17.75 *p* = 0.012] and admission blood glucose [9.03 ± 2.77 vs. 7.42 ± 2.22 *p* < 0.001], and hypertension [66 (89.2%) vs. 77 (62.1%) *p* < 0.001] compared to the UR group.

**Table 1:** Comparison of clinical characteristics between the two groups.

	All (198)	UR (*n* = 124)	FR (*n* = 74)	*t*/*χ* 2	*p*
Age, years, mean ± SD	70.54 ± 12.12	70.26 ± 12.94	70.71 ± 11.67	0.254	0.800
Sex				0.065	0.799
Man (*n*, %)	120 (60.6%)	76 (49.3%)	44 (59.5%)		
Woman (*n*, %)	78 (39.4%)	48 (38.7%)	30 (40.5%)		
Smoking (*n*, %)	60 (30.3%)	40 (32.3%)	20 (27%)	0.6	0.438
Past medical history					
Prior stroke, *N* (%)	34 (17.2%)	22 (17.7%)	12 (16.2%)	0. 076	0.783
Hyperlipidemia, *N* (%)	6 (3.0%)	2 (1.6%)	4 (5.4%)	2.268	0.132
Atrial fibrillation, *N* (%)	69 (34.8%)	41 (34.1%)	28 (37.8%)	0.465	0.495
Diabetes mellitus, *N* (%)	40 (20.2%)	21 (16.9%)	19 (25.7%)	2.196	0.138
Coronary heart disease, *N* (%)	31 (15.7%)	20 (16.1%)	11 (14.9%)	0.056	0.813
Hypertension, *N* (%)	144 (72.2%)	77 (62.1%)	66 (89.2%)	16.956	<0.001
Admission NIHSS, [M (Q1, Q3)]	18 [13.0–21.0]	15.0 [11.0–20.0]	20 [17.0–29.0]	5.979	<0.001
Alcohol, *N* (%)	67 (33.8%)	45 (36.3%)	22 (29.7%)	0.891	0.345
Stoke subtype by TOAST criteria					
LAA	100 (50.5%)	64 (51.6%)	36 (48.6%)	1.812	0.612
CE	87 (43.9%)	53 (42.7%)	34 (45.9%)		
SAO	3 (1.5%)	1 (0.8%)	2 (2.7%)		
Other causes	8 (4.1%)	6 (4.8%)	2 (2.7%)		
Stroke workflow times					
DPT [min, M (Q1, Q3)]	87.5 (67, 110)	83 (51.5, 102.75)	97 (79.75, 125.25)	3.239	0.001
OPT [min, M (Q1, Q3)]	349 (256, 506)	349 (256, 521.75)	349.5 (266.25, 446)	0.579	0.563
DRT [min, M (Q1, Q3)]	123 (90, 157.75)	116 (83.25, 144)	137 (105.75, 178.5)	3.005	0.003
Biochemical variables					
Admission SBP (x̄ ± s, mmHG)	148.80 ± 24.25	147.44 ± 23.31	151.09 ± 24.17	1.027	0.305
Admission DBP (x̄ ± s, mmHG)	89.55 ± 17.78	87.10 ± 17.75	93.64 ± 17.18	2.535	0.012
Admission glucose (x̄ ± s, mmol/L)	8.92 ± 2.56	7.42 ± 2.22	9.03 ± 2.77	4.482	<0.001
TG (x̄ ± s, mmol/L)	1.26 ± 0.83	1.19 ± 0.72	1.38 ± 0.99	1.428	0.156
TC (x̄ ± s, mmol/L)	3.87 ± 0.92	3.91 ± 0.84	3.78 ± 1.04	0.906	0.367
LDL (x̄ ± s, mmol/L)	2.12 ± 0.76	2.11 ± 0.71	2.13 ± 0.85	0.117	0.833
Ischemic penumbra (x̄ ± s, ml)	118.56 ± 84.64	108.63 ± 84.29	135.19 ± 83.15	2.156	0.032
Ischemic core (x̄ ± s, ml)	27.39 ± 26.81	22.48 ± 22.35	37.31 ± 30.66	3.872	<0.001

LAA, large-artery atherosclerosis; CE, cardioembolism; SAO, small-artery occlusion; DPT, Door-to-Puncture Time; OPT, Onset-to-Puncture Time; DRT, Door-to-Recanalization Time; TG, triglyceride; TC, total cholesterol; LDL, Low Density Lipoprotein; mTICI, modified Thrombolysis in Cerebral Infarction; FR, Univariate analyses of demographics based on futile recanalization.

### Univariate analysis of risk factors

A total of 198 patients were included, with 74 cases (37.37%) FR and 124 cases (62.63%) without FR. The univariate analysis revealed statistically significant differences in hypertension, Baseline NIHSS, DPT, DRT, Admission DBP, Admission blood glucose, ischemic penumbra and ischemic core between the two groups (*p* < 0. 05). The remaining indicators were not statistically significant between the two groups (Table 2).

**Table 2:** Predictors of futile recanalization.

Factors	B-coefficient	Standard error	Wald χ 2	*p*-value	OR	95% CI
Hypertension	1.453	0.516	7.921	0.005	4.752	1.554–11.754
Admission NIHSS	0.131	0.029	20.720	<0.001	1.150	1.077–1.206
Admission DBP	0.027	0.011	6.395	0.011	1.035	1.006–1.050
Admission blood glucose	0.192	0.086	4.932	0.026	1.250	1.023–1.434
Ischemic core	0.024	0.008	9.357	0.002	1.022	1.009–1.040
DPT	0.011	0.004	8.497	0.004	1.011	1.005–1.018

### Multi-factor logistic regression analysis of risk factors for poor prognosis

With the presence of unfavorable outcome occurred as the dependent variable (yes as signed = 1, no assigned = 0), indicators that differed on univariate analysis, including hypertension, Baseline NIHSS, DPT, DRT, Admission DBP, Admission blood glucose, ischemic penumbra and ischemic core as independent variables according to the relevant literature were subjected to binary logistic regression analysis with the entry method selected “Forward LR.” Results revealed that greater baseline NIHSS score, admission DBP and blood glucose; bigger ischemic core; and longer DPT were risk factors for the presence of adverse prognosis (all *p* < 0.05) (Table 3).

**Table 3:** Analysis of the ability of each index to predict futile recanalization.

Item	AUC	SE	*p* value	95% CI	Sensitivity	Specificity
Baseline NIHSS score	0.737	0.036	<0.001	0.668–0.807	0.649	0.351
Admission DBP	0.605	0.039	<0.001	0.492–0.659	0.662	0.338
Admission blood glucose	0.715	0.038	<0.001	0.641–0.790	0.784	0.216
Ischemic core	0.67	0.040	<0.001	0.591–0.749	0.581	0.419
DPT	0.658	0.039	<0.001	0.581–0.739	0.784	0.216
Combine model	0.851	0.27	<0.001	0.798–0.904	0.743	0.839

### Analysis of the ability to predict the risk of FR

The FR was used as the status variable (yes = 1, no = 0). Logistic regression was first used to construct a model of baseline NIHSS score, admission DBP, admission blood glucose, ischemic core and DPT separately. ROC analysis was performed that the area under the curve (AUC) of the baseline NIHSS score, admission DBP, admission blood glucose, ischemic core and DPT for predicting the FR were 0.737 (95% CI, 0.668–0.807), 0.605 (95% CI, 0.492–0.659), 0.715 (95% CI, 0.641–0.790), 0.67 (95% CI, 0.591–0.749), 0.658 (95% CI, 0.581–0.739). Then, using logistic regression to construct a combined model of the included 5 risk factors, which had the highest AUC (0.852; 95% CI, 0.798–0.904), a specificity of 83.9%, a sensitivity of 74.3% and a Jorden index of 0.582 for predicting the FR, suggesting that the best ability to predict the risk of FR occurred when the baseline NIHSS score was 18.5, admission DBP was 87.5 mmHg, admission blood glucose was 8.515 mmol/L, ischemic core 28.5 mL and DPT was 78.5 min.

## Discussion

The elevated incidence, mortality, and disability rates correlated with AIS impose a substantial burden on patients, society, and the nation (29). The primary goal of AIS treatment is to restore blood flow and prevent ongoing neuronal injury. Revascularization efforts rely on intravenous thrombolysis and MT etc., which serve as the foundational approaches for managing AIS (26). FR has been reported in up to 48.7% of patients following MT and is a key factor influencing their prognosis (30). Recognizing the risk factors for FR is critical for optimizing postoperative functional outcomes in patients. In this study involving 198 patients, the main results of our study were baseline NIHSS, admission blood glucose, elevation in DBP, ischemic core and DPT which were associated with FR in AIS-LVO patients following MT. In our findings, we identified five independent variables associated with FR. Among them, two variables are non-modifiable: the ischemic core and baseline NIHSS. The other three variables are modifiable: DPT, diastolic blood pressure, and baseline blood glucose.

Several previous studies reported that large ischemic cores are strong poor prognostic factors for AIS. Campbell et al. (31) reported that in patients undergoing mechanical thrombectomy and intravenous thrombolysis, a 10 mL increase in ischemic core volume is associated with worse functional outcomes. Yang et al. (32). demonstrated that AIS patients with an ischemic core volume greater than 90 mL exhibited poor prognoses and were less likely to benefit from MT. A study conducted in Japan by Yoshimoto et al. (33). observed that patients with an ischemic core volume between 70 and 100 mL could potentially benefit from MT. However, they proposed that the maximum threshold for therapeutic efficacy might be as high as 120 mL. Our results align with previous studies, demonstrating that each 1 mL increase in ischemic core volume is associated with an odds ratio for FR of 1.022 (95% CI: 1.009–1.040). Although ischemic core volume is widely utilized as an imaging biomarker in patients undergoing MT (34, 35), it is subject to several limitations, including variability in CT perfusion imaging software algorithms and inter-observer variability (36–39). The formation of the ischemic core in acute ischemic stroke (AIS) is predominantly attributed to diminished cerebral perfusion, resulting in irreversible ischemic damage or even necrosis of affected tissue (40). Consequently, it is essential to employ therapeutic interventions such as intravenous thrombolysis or mechanical thrombectomy (MT) to restore perfusion in the ischemic region and mitigate further neurological damage (41).

This study utilized the baseline NIHSS score at admission to assess the severity of stroke in AIS patients with FR. Our finding of a higher NIHSS score and its association with the FR is consistent with several previous studies which evaluated the prognostic factors of outcome after MT. A study conducted by Heitkamp et al. (42). analyzing 13,082 AIS patients treated with mechanical MT reported that each 1-point increment in the admission NIHSS score was associated with an adjusted odds ratio for FR of 1.09 (95% CI: 1.05–1.14). A retrospective study by Lee et al. (43) proposed that the therapeutic benefits of endovascular treatment are positively correlated with stroke severity. The probability of FR was observed to be 20.9% in patients with an NIHSS score ≤5, 34.6% in those with an NIHSS score of 6–10, 58.9% in individuals with an NIHSS score of 11–20, and 63.8% in those with an NIHSS score ≥20.

Our study confirmed the correlation between door-to-puncture time (DPT) delay and FR. The time interval between stroke onset and the start of MT has been proposed as a critical factor influencing the success of positive trials (44). However, our findings demonstrate that patients with FR exhibited significantly prolonged DPT in comparison to those achieving effective recanalization. The median DPT (min) in patients with FR was significantly higher compared to patients with effective recanalization in our cohort, with respective values of 83 (51.5, 102.75) vs. 97 (79.75, 125.25) (*p* = 0.001). Saver et al. (45) suggested that if their research were expanded to a larger population, the DPT time of 1,000 AIS patients could potentially be reduced by 15 min. Additionally, it is estimated that approximately 39 patients would experience a decrease in disability level after 3 months, with 25 of them achieving functional independence (mRS 0–2). This study provides additional evidence regarding the association between treatment time and the benefit of endovascular reperfusion. On the other hand, this study found no significant in OPT. This is mainly due to differences in the reliability of recording the time of stroke onset and how arrival times at the emergency department are recorded. Typically, arrival times at the emergency department are accurately documented in patients’ medical records as Door-to-Puncture (DPT) times. In contrast, the exact time of a stroke onset is often difficult to determine or record. Some patients may experience symptoms while sleeping, making it difficult to determine the actual time when symptoms appeared. Additionally, some patients with neurological deficits may not be able to accurately observe or report specific times when symptoms occurred (45, 46).

Another important finding of our study is that higher blood glucose levels correlated with an increased likelihood of FR. It is commonly observed that patients with AIS often exhibit elevated blood glucose levels. The study (47), which included 302 patients with AIS, revealed an independent association between elevated baseline glucose levels and futile reperfusion in patients (blood glucose level [OR, 1.121; 95% CI 1.027–1.223; *p* = 0.010]). The meta-analysis conducted by Shen et al. (48) covered 5,099 patients in 17 studies (SMD = 0.313, 95% CI: [0.217; 0.409], *p* < 0.001). The authors pointed out that baseline blood glucose was associated with FR regardless of whether it was anterior circulation occlusion or mixed occlusion in AIS. Furthermore, we need to pay attention to the increase in stress-induced hyperglycemia in non-diabetic patients. This is caused by a series of reactions triggered by stressors such as trauma and AIS, which in turn stimulate the activation of the sympathetic nerve and the pituitary–adrenal axis. Higher concentrations of catecholamines, cortisol, and inflammatory factors mainly cause a transient increase in blood glucose through the process of gluconeogenesis (49, 50). To date, no empirical evidence has demonstrated a significant association between tight blood glucose control and improved outcomes following AIS. In conclusion, the rise in baseline blood glucose upon admission can, to a certain extent, reflect the stress status of patients. Nevertheless, its drawback lies in the failure to consider the influence of background blood glucose, such as factors like diet and rest schedule, thereby affecting the final treatment outcome. Hence, it is suggested that medical staff adopt multiple approaches to detect and manage the blood glucose status of patients, such as indicators like stress-induced hyperglycemia, while also taking into account the background glucose conditions of patients.

Interestingly, this study identified an association between baseline DBP levels and FR. however, the average baseline SBP was higher in FR patients (although not statistically significant), which conflicts with the previous cognition that both low and high SBP are associated with poor prognosis in previous studies (51, 52). In a prospective cohort study, Sphoie et al. (52). indicated that among 3,180 AIS patients who underwent MT, the relationship between baseline systolic blood pressure (SBP) and diastolic blood pressure (DBP) and poor functional prognosis exhibited a J-shaped curve. The inflection points of both were the median values of 150 mmHg and 81 mmHg, respectively. When SBP and DBP exceeded the median values, the odds of poor functional outcomes and mortality at 90 days increased. While our findings imply that very baseline DBP lowering may be beneficial in AIS patients for MT, it is essential to point out that our results do not prove a direct causal relationship between high DBP and FR. Meanwhile, in the management of blood pressure for AIS patients, both SBP and DBP should be taken into account. Standard blood pressure management should be implemented, which can lead to better 90-day functional outcomes (53, 54). The differences in baseline SBP and DBP in this study might be attributed to the relatively small sample size of the study population (*n* = 199).

Since AIS patients are at a higher clinical risk after MT, predicting the occurrence of FR can offer valuable guidance for AIS after MT. In addition, this is the first study to integrate baseline NIHSS scores, admission blood glucose, admission DBP, ischemic core and DPT as predictors. The purpose of this model is to provide prognostic guidance for AIS-LVO patients, aiming to optimize targeted strategies such as blood pressure management and reduced door-to-puncture time (DPT) for enhanced care efficiency—not to restrict access to thrombectomy. It is of great significance to develop a risk prediction model that conforms to the characteristics of post-MT FR in AIS patients in China, enhances clinical efficiency, and reduces the harm caused by post-MT FR in AIS patients.

In this study, a combined model of baseline NIHSS scores, admission blood glucose, admission DBP, ischemic core and DPT was constructed by logistic regression analysis. Subsequently, the predictive ability was evaluated by plotting the area under the ROC curve. It was discovered that the predictive efficacy of the combined model was superior to the predictive value of each of the five factors individually when the baseline NIHSS score > 18.5, admission DBP > 87.5 mmHg, admission blood glucose > 8.515 mmol/L, ischemic core >28.5 mL and DPT > 78.5 min. The risk of FR after MT with AIS was greater when these occurred.

### Limitations

Nevertheless, a single-center retrospective study was one of the limitations in our study, the sample size of 199 patients is relatively small, which might have selection bias. Also limiting the generalizability of its findings to broader populations with different demographic or healthcare system characteristics. Patient management protocols, resource availability, and population characteristics (e.g., genetics, stroke risk factors) can vary significantly across regions and institutions. This may lead to results that are not universally applicable. Larger-scale studies are requisite to validate these results and guarantee their application to a broader population. On the one hand, the baseline blood glucose upon admission may be influenced by various factors. Secondly, the NIHSS score has certain limitations in posterior circulation cerebral infarction. Therefore, future studies could use stress blood glucose data including HBA1c and require more adjusted or upgraded NIHSS scores to describe post-circulation cerebral infarction in AIS patients. Additionally, hospital ethics review rules and differences in how patient outcomes are measured between departments have made it hard to share important patient information—like blood pressure during surgery and ASPECTS scores (a type of brain scan assessment). Another issue is cultural differences between Eastern and Western traditions. Patients with poor recovery results often avoid follow-up visits or sharing private details due to personal or family concerns, which limits the collection of key data such as death rates, symptomatic brain bleeding, and blood flow support in the brain. These challenges should be carefully looked into in larger future studies to improve data sharing and patient participation.

## Conclusion

In this study, a combined model of baseline NIHSS scores, admission blood glucose, admission DBP, ischemic core and DPT was constructed by logistic regression analysis. Subsequently, the predictive ability was evaluated by plotting the area under the ROC curve. The indicators employed in this study are readily accessible in clinical practice. The combined model demonstrates high diagnostic efficacy and can be utilized to assess the functional recovery of patients with acute ischemic stroke after mechanical thrombectomy, presenting practical value for guiding clinical treatment and rehabilitation.

## Data Availability

The datasets presented in this article are not readily available because of patients’ personal medical information and privacy. Requests to access these datasets should be directed to ganying3304@163.com.

## References

[1] Roth GA Mensah GA Johnson CO Addolorato G Ammirati E Baddour LM. Global burden of cardiovascular diseases and risk factors, 1990-2019: update from the GBD 2019 study. J Am Coll Cardiol. (2020) 76:2982–3021. doi: 10.1016/j.jacc.2020.11.010 33309175 PMC7755038

[2] Wang YJ Li ZX Gu HQ Zhai Y Zhou Q Jiang Y. China stroke statistics: an update on the 2019 report from the National Center for healthcare quality Management in Neurological Diseases, China National Clinical Research Center for neurological diseases, the Chinese Stroke Association, National Center for chronic and non-communicable disease control and prevention, Chinese Center for Disease Control and Prevention and institute for global neuroscience and stroke collaborations. Stroke Vasc Neurol. (2022) 7:415–50. doi: 10.1136/svn-2021-001374, PMID: 35443985 PMC9614174

[3] Mendelson SJ Prabhakaran S. Diagnosis and Management of Transient Ischemic Attack and Acute Ischemic Stroke: a review. JAMA. (2021) 325:1088–98. doi: 10.1001/jama.2020.26867, PMID: 33724327

[4] Jauch EC Saver JL Adams HP Jr Bruno A Connors JJ Demaerschalk BM. Guidelines for the early management of patients with acute ischemic stroke: a guideline for healthcare professionals from the American Heart Association/American Stroke Association. Stroke. (2013) 44:870–947. doi: 10.1161/STR.0b013e318284056a, PMID: 23370205

[5] Levy EI Siddiqui AH Crumlish A Snyder KV Hauck EF Fiorella DJ. First Food and Drug Administration-approved prospective trial of primary intracranial stenting for acute stroke: SARIS (stent-assisted recanalization in acute ischemic stroke). Stroke. (2009) 40:3552–6. doi: 10.1161/STROKEAHA.109.561274 19696415

[6] Powers WJ Rabinstein AA Ackerson T Adeoye OM Bambakidis NC Becker K. 2018 guidelines for the early Management of Patients with Acute Ischemic Stroke: a guideline for healthcare professionals from the American Heart Association/American Stroke Association. Stroke. (2018) 49:e46–e110. doi: 10.1161/STR.0000000000000158, PMID: 29367334

[7] Xiong Y Wakhloo AK Fisher M. Advances in acute ischemic stroke therapy. Circ Res. (2022) 130:1230–51. doi: 10.1161/CIRCRESAHA.121.319948 35420919

[8] Campbell BCV Donnan GA Lees KR Hacke W Khatri P Hill MD. Endovascular stent thrombectomy: the new standard of care for large vessel ischaemic stroke. Lancet Neurol. (2015) 14:846–54. doi: 10.1016/S1474-4422(15)00140-4 26119323

[9] Bracard S Ducrocq X Mas JL Soudant M Oppenheim C Moulin T. Mechanical thrombectomy after intravenous alteplase versus alteplase alone after stroke (THRACE): a randomised controlled trial. Lancet Neurol. (2016) 15:1138–47. doi: 10.1016/S1474-4422(16)30177-6 27567239

[10] Campbell BC Mitchell PJ Kleinig TJ Dewey HM Churilov L Yassi N. Endovascular therapy for ischemic stroke with perfusion-imaging selection. N Engl J Med. (2015) 372:1009–18. doi: 10.1056/NEJMoa1414792, PMID: 25671797

[11] Saver JL Goyal M Bonafe A Diener HC Levy EI Pereira VM. Stent-retriever thrombectomy after intravenous t-PA vs. t-PA alone in stroke. N Engl J Med. (2015) 372:2285–95. doi: 10.1056/NEJMoa1415061, PMID: 25882376

[12] Jovin TG Chamorro A Cobo E de Miquel MA Molina CA Rovira A. Thrombectomy within 8 hours after symptom onset in ischemic stroke. N Engl J Med. (2015) 372:2296–306. doi: 10.1056/NEJMoa1503780, PMID: 25882510

[13] Mocco J Zaidat OO von Kummer R Yoo AJ Gupta R Lopes D. Aspiration thrombectomy after intravenous alteplase versus intravenous alteplase alone. Stroke. (2016) 47:2331–8. doi: 10.1161/STROKEAHA.116.013372 27486173

[14] Berkhemer OA Fransen PS Beumer D van den Berg LA Lingsma HF Yoo AJ. A randomized trial of intraarterial treatment for acute ischemic stroke. N Engl J Med. (2015) 372:11–20. doi: 10.1056/NEJMoa1411587 25517348

[15] Turc G Bhogal P Fischer U Khatri P Lobotesis K Mazighi M. European stroke organisation (ESO) - European Society for Minimally Invasive Neurological Therapy (ESMINT) guidelines on mechanical Thrombectomy in acute ischemic stroke. J Neurointerv Surg. (2023) 15:e8. doi: 10.1136/neurintsurg-2018-014569, PMID: 30808653

[16] Goyal M Demchuk AM Menon BK Eesa M Rempel JL Thornton J. Randomized assessment of rapid endovascular treatment of ischemic stroke. N Engl J Med. (2015) 372:1019–30. doi: 10.1056/NEJMoa1414905, PMID: 25671798

[17] Badhiwala JH Nassiri F Alhazzani W Selim MH Farrokhyar F Spears J. Endovascular thrombectomy for acute ischemic stroke: a meta-analysis. JAMA. (2015) 314:1832–43. doi: 10.1001/jama.2015.13767 26529161

[18] Goyal M Menon BK van Zwam WH Dippel DW Mitchell PJ Demchuk AM. Endovascular thrombectomy after large-vessel ischaemic stroke: a meta-analysis of individual patient data from five randomised trials. Lancet. (2016) 387:1723–31. doi: 10.1016/S0140-6736(16)00163-X, PMID: 26898852

[19] Nogueira RG Jadhav AP Haussen DC Bonafe A Budzik RF Bhuva P. Thrombectomy 6 to 24 hours after stroke with a mismatch between deficit and infarct. N Engl J Med. (2018) 378:11–21. doi: 10.1056/NEJMoa1706442, PMID: 29129157

[20] Kaesmacher J Dobrocky T Heldner MR Bellwald S Mosimann PJ Mordasini P. Systematic review and meta-analysis on outcome differences among patients with TICI2b versus TICI3 reperfusions: success revisited. J Neurol Neurosurg Psychiatry. (2018) 89:910–7. doi: 10.1136/jnnp-2017-317602, PMID: 29519899 PMC6109240

[21] van Horn N Kniep H Leischner H McDonough R Deb-Chatterji M Broocks G. Predictors of poor clinical outcome despite complete reperfusion in acute ischemic stroke patients. J Neurointerv Surg. (2021) 13:14–8. doi: 10.1136/neurintsurg-2020-015889, PMID: 32414889

[22] Shahid AH Abbasi M Larco JLA Madhani SI Liu Y Brinjikji W. Risk factors of futile recanalization following endovascular treatment in patients with large-vessel occlusion: systematic review and meta-analysis. Stroke. (2022) 2:e000257. doi: 10.1161/SVIN.121.000257 PMC12778831 41582963

[23] Kniep H Meyer L Broocks G Bechstein M Heitkamp C Winkelmeier L. Thrombectomy for M2 occlusions: predictors of successful and futile recanalization. Stroke. (2023) 54:2002–12. doi: 10.1161/STROKEAHA.123.043285 37439204

[24] Peduzzi P Concato J Feinstein AR Holford TR. Importance of events per independent variable in proportional hazards regression analysis. II. Accuracy and precision of regression estimates. J Clin Epidemiol. (1995) 48:1503–10. doi: 10.1016/0895-4356(95)00048-8, PMID: 8543964

[25] Adams HP Jr Bendixen BH Kappelle LJ Biller J Love BB Gordon DL. Classification of subtype of acute ischemic stroke. Definitions for use in a multicenter clinical trial. TOAST. Trial of org 10172 in acute stroke treatment. Stroke. (1993) 24:35–41. doi: 10.1161/01.STR.24.1.35, PMID: 7678184

[26] Herpich F Rincon F. Management of Acute Ischemic Stroke. Crit Care Med. (2020) 48:1654–63. doi: 10.1097/CCM.0000000000004597, PMID: 32947473 PMC7540624

[27] Zaidat OO Yoo AJ Khatri P Tomsick TA von Kummer R Saver JL. Recommendations on angiographic revascularization grading standards for acute ischemic stroke: a consensus statement. Stroke. (2013) 44:2650–63. doi: 10.1161/STROKEAHA.113.001972, PMID: 23920012 PMC4160883

[28] Albers GW Marks MP Kemp S Christensen S Tsai JP Ortega-Gutierrez S. Thrombectomy for stroke at 6 to 16 hours with selection by perfusion imaging. N Engl J Med. (2018) 378:708–18. doi: 10.1056/NEJMoa1713973, PMID: 29364767 PMC6590673

[29] Zhou P Liu J Wang L Feng W Cao Z Wang P. Association of Small Dense low-Density Lipoprotein Cholesterol with stroke risk, severity and prognosis. J Atheroscler Thromb. (2020) 27:1310–24. doi: 10.5551/jat.53132, PMID: 32062644 PMC7840160

[30] Deng G Xiao J Yu H Chen M Shang K Qin C. Predictors of futile recanalization after endovascular treatment in acute ischemic stroke: a meta-analysis. J Neurointerv Surg. (2022) 14:881–5. doi: 10.1136/neurintsurg-2021-017963, PMID: 34544824

[31] Campbell BCV Majoie C Albers GW. Penumbral imaging and functional outcome in patients with anterior circulation ischaemic stroke treated with endovascular thrombectomy versus medical therapy: a meta-analysis of individual patient-level data. Lancet Neurol. (2019) 18:46–55. doi: 10.1016/S1474-4422(18)30314-4, PMID: 30413385

[32] Yang H Lin D Lin X Wu Y Yi T Chen W. Outcomes and CT perfusion thresholds of mechanical Thrombectomy for patients with large ischemic Core lesions. Front Neurol. (2022) 13:856403. doi: 10.3389/fneur.2022.856403, PMID: 35720105 PMC9198314

[33] Yoshimoto T Inoue M Tanaka K Kanemaru K Koge J Shiozawa M. Identifying large ischemic core volume ranges in acute stroke that can benefit from mechanical thrombectomy. J Neurointerv Surg. (2021) 13:1081–7. doi: 10.1136/neurintsurg-2020-016934, PMID: 33323502 PMC8606466

[34] Koopman MS Hoving JW Kappelhof M Berkhemer OA Beenen LFM van Zwam WH. Association of Ischemic Core Imaging Biomarkers with Post-Thrombectomy Clinical Outcomes in the MR CLEAN registry. Front Neurol. (2021) 12:771367. doi: 10.3389/fneur.2021.771367, PMID: 35082746 PMC8784730

[35] Broocks G Kniep H Schramm P Hanning U Flottmann F Faizy T. Patients with low Alberta stroke program early CT score (ASPECTS) but good collaterals benefit from endovascular recanalization. J Neurointerv Surg. (2020) 12:747–52. doi: 10.1136/neurintsurg-2019-015308, PMID: 31772043

[36] Vagal A Wintermark M Nael K Bivard A Parsons M Grossman AW. Automated CT perfusion imaging for acute ischemic stroke: pearls and pitfalls for real-world use. Neurology. (2019) 93:888–98. doi: 10.1212/WNL.0000000000008481, PMID: 31636160

[37] Austein F Riedel C Kerby T Meyne J Binder A Lindner T. Comparison of perfusion CT software to predict the final infarct volume after Thrombectomy. Stroke. (2016) 47:2311–7. doi: 10.1161/STROKEAHA.116.013147, PMID: 27507864

[38] Gupta AC Schaefer PW Chaudhry ZA Leslie-Mazwi TM Chandra RV González RG. Interobserver reliability of baseline noncontrast CT Alberta stroke program early CT score for intra-arterial stroke treatment selection. AJNR Am J Neuroradiol. (2012) 33:1046–9. doi: 10.3174/ajnr.A2942, PMID: 22322602 PMC8013224

[39] Berkhemer OA Jansen IG Beumer D. Collateral status on baseline computed tomographic angiography and intra-arterial treatment effect in patients with proximal anterior circulation stroke. Stroke. (2016) 47:768–76. doi: 10.1161/STROKEAHA.115.011788, PMID: 26903582

[40] Vagal A Menon BK Foster LD Livorine A Yeatts SD Qazi E. Association between CT angiogram collaterals and CT perfusion in the interventional Management of Stroke III trial. Stroke. (2016) 47:535–8. doi: 10.1161/STROKEAHA.115.011461, PMID: 26658448 PMC4729636

[41] Uniken Venema SM Dankbaar JW van der Lugt A Dippel DWJ van der Worp HB. Cerebral collateral circulation in the era of reperfusion therapies for acute ischemic stroke. Stroke. (2022) 53:3222–34. doi: 10.1161/STROKEAHA.121.037869 35938420

[42] Heitkamp C Heitkamp A Winkelmeier L Thaler C Flottmann F Schell M. Predictors of futile recanalization in ischemic stroke patients with low baseline NIHSS. Int J Stroke. (2024) 19:1102–12. doi: 10.1177/17474930241264737, PMID: 38888031 PMC11590392

[43] Lee SH Kim BJ Han MK Park TH Lee KB. Futile reperfusion and predicted therapeutic benefits after successful endovascular treatment according to initial stroke severity. BMC Neurol. (2019) 19:11. doi: 10.1186/s12883-019-1237-2, PMID: 30646858 PMC6332890

[44] Hacke W. Interventional thrombectomy for major stroke--a step in the right direction. N Engl J Med. (2015) 372:76–7. doi: 10.1056/NEJMe1413346 25517349

[45] Saver JL Goyal M van der Lugt A Menon BK Majoie CBLM Dippel DW. Time to treatment with endovascular Thrombectomy and outcomes from ischemic stroke: a meta-analysis. JAMA. (2016) 316:1279–88. doi: 10.1001/jama.2016.13647, PMID: 27673305

[46] Almekhlafi MA Goyal M Dippel DWJ Majoie CBLM Campbell BCV. Healthy life-year costs of treatment speed from arrival to endovascular Thrombectomy in patients with ischemic stroke: a meta-analysis of individual patient data from 7 randomized clinical trials. JAMA Neurol. (2021) 78:709–17. doi: 10.1001/jamaneurol.2021.1055, PMID: 33938914 PMC8094030

[47] Su M Zhou Y Chen Z Pu M Li Z Du H. Cystatin C predicts futile recanalization in patients with acute ischemic stroke after endovascular treatment. J Neurol. (2022) 269:966–72. doi: 10.1007/s00415-021-10680-w, PMID: 34226965

[48] Shen H Killingsworth MC Bhaskar SMM. Comprehensive Meta-analysis of futile recanalization in acute ischemic stroke patients undergoing endovascular Thrombectomy: prevalence, factors, and clinical outcomes. Life. (2023) 13:1965. doi: 10.3390/life13101965, PMID: 37895347 PMC10608522

[49] Dungan KM Braithwaite SS Preiser J-C. Stress hyperglycaemia. Lancet. (2009) 373:1798–807. doi: 10.1016/S0140-6736(09)60553-5, PMID: 19465235 PMC3144755

[50] Genceviciute K Göldlin MB Kurmann CC Mujanovic A Meinel TR Kaesmacher J. Association of diabetes mellitus and admission glucose levels with outcome after endovascular therapy in acute ischaemic stroke in anterior circulation. Eur J Neurol. (2022) 29:2996–3008. doi: 10.1111/ene.15456, PMID: 35719010 PMC9544025

[51] Anadani M Arthur AS Alawieh A Orabi Y Alexandrov A Goyal N. Blood pressure reduction and outcome after endovascular therapy with successful reperfusion: a multicenter study. J Neurointerv Surg. (2020) 12:932–6. doi: 10.1136/neurintsurg-2019-015561, PMID: 31806668 PMC7998040

[52] van den Berg SA Uniken Venema SM Mulder M. Admission blood pressure in relation to clinical outcomes and successful reperfusion after endovascular stroke treatment. Stroke. (2020) 51:3205–14. doi: 10.1161/STROKEAHA.120.029907, PMID: 33040702 PMC7587243

[53] Jiang S Zhou Y Zhou Y Huang G. Intensive blood pressure management for ischemic stroke patients following endovascular thrombectomy: a meta-analysis of randomized controlled trials. BMC Neurol. (2024) 24:469. doi: 10.1186/s12883-024-03976-7, PMID: 39627722 PMC11613891

[54] Zhou Y Chen Z Fang J Huang G. Blood pressure targets for acute ischemic stroke patients following endovascular thrombectomy: a meta-analysis. Clin Neurol Neurosurg. (2023) 231:107835. doi: 10.1016/j.clineuro.2023.107835, PMID: 37354634

